# Sex differences in the inference and perception of causal relations within a video game

**DOI:** 10.3389/fpsyg.2014.00926

**Published:** 2014-08-22

**Authors:** Michael E. Young

**Affiliations:** Department of Psychological Sciences, Kansas State UniversityManhattan, KS, USA

**Keywords:** causal inference, causal perception, learning, video games, sex differences

## Abstract

The learning of immediate causation within a dynamic environment was examined. Participants encountered seven decision points in which they needed to choose, which of three possible candidates was the cause of explosions in the environment. Each candidate was firing a weapon at random every few seconds, but only one of them produced an immediate effect. Some participants showed little learning, but most demonstrated increases in accuracy across time. On average, men showed higher accuracy and shorter latencies that were not explained by differences in self-reported prior video game experience. This result suggests that prior reports of sex differences in causal choice in the game are not specific to situations involving delayed or probabilistic causal relations.

## INTRODUCTION

### CAUSAL DISCOVERY AND GENERALIZATION CONTRIBUTION TO FRONTIERS

Throughout our lifetime, we are faced with a complex web of causal relationships that drive the evolution of our world. Physical objects interact to produce changes in the physical environment. Changes in that physical environment produce changes in the organisms living within that environment. Those organisms produce changes in one another as they interact through direct physical contact, auditory exchanges, olfactory cues, or other senses. These same organisms bring about changes in their physical environments. This network of causation determines the future, and an organism’s success is directly affected by its ability to prepare for this future. Identification of causal relations permits a person, dog, or fly to improve its future by carrying an umbrella, salivating, or avoiding a looming fly swatter.

Some causal relationships seem to be readily apprehended or perceived whereas others require a process of discovery. Research on the former is presented as the study of causal perception (e.g., Michotte, 1946/1963; Schlottmann and Surian, 1999; Scholl and Nakayama, 2004) whereas research on the latter is presented as the study of causal learning or inference (e.g., Shanks et al., 1996; Wasserman et al., 1996). This dichotomy assumes a natural and categorical distinction that is less clear when people’s behavior is examined. Causal perception is often assumed when causal judgments are automatic – when there is direct physical and immediate interaction between two objects a single observation is sufficient to elicit a confident judgment of causation. Causal learning or inference is assumed when a series of experiences are necessary in order to discover the relations.

However, some relationships that seem to be directly perceived also have been shown to be affected by experience (e.g., Gruber et al., 1957; Powesland, 1959; Young et al., 2005), and others that are normally learned can readily generalize to a novel situation thus producing instantaneous causal judgment (Glautier, 2004; White, 2006). Furthermore, there may be significant individual differences in causal judgment which gives rise to questions regarding their origin (genetic or ontogenetic). Unfortunately, the role of experience in causal perception is often not apparent because studies frequently do not report how these judgments evolve over time nor do they report individual differences in these judgments. This lack of analysis is also often present in my own work on causality, and this manuscript represents a step toward filling this gap.

In a program involving causal perception and learning within a video game, my colleagues and I routinely use the first block of the game to familiarize people with the gaming environment and to ensure understanding of the task. Decisions during this block involve a perfect contingency (i.e., no probabilistic causality) and immediate causation. Participants are faced with trios of candidate causes (“orcs” or monsters shooting weapons) of a distal explosion on a building that they are tasked to protect. One of the orcs is the cause of these explosions, and it is the participant’s task to identify this orc and eliminate it. In all of our reported experiments, we ignore behavior during this initial block of decisions and only report behavior once delays, probabilistic relations, and other independent variables are introduced. In the present project, this previously ignored data will finally receive some attention.

During initial exposure to causal relations in the game, judgment is assumed to be based on causal perception because very little inference is required. If an orc fires and an explosion immediately follows, the cause should be quite evident. However, there are two factors that make this apparently easy decision a bit more difficult. First, there are three causal candidates to choose from, and it is possible that two of them may fire in close temporal proximity simply by chance (their firing is scheduled to repeat at random intervals). Under these conditions, it would be necessary to observe a second shot by these candidates to de-confound their actions and consequences. Second, this situation is a novel one. Although people likely assume that the firing of a weapon would cause an immediate consequence, some participants may be reluctant to assume that relations observed on the firing range or television would necessarily translate to this gaming environment. Because we have not analyzed behavior during this initial exposure, it was not known if people’s behavior evolved during this period.

In examining behavior during initial exposure, I was also interested in revisiting the evolution of individual differences. In our prior work, we have often reported that men have higher accuracies and take less time to make their decisions, but the accuracy difference can sometimes be accounted for by differences in self-reported video game experience by women and men (Young and Nguyen, 2009). Are these performance differences evident at the outset even when the causal relationship is perfect (100% and immediate) or do they only emerge later in the game when introducing delayed or probabilistic causation? If there are differences during this initial period, they could arise due to different intercepts, indicating different preparedness for the task, or different learning rates as participants adapt to the task. In the near absence of prior research on sex differences in causal perception and judgment, it is difficult to form hypotheses. Although the small amount of data available (e.g., Shaklee and Hall, 1983; Wasserman et al., 1983, Wasserman and Neunaber, 1986) suggest the possible presence of sex differences in non-gaming causality tasks, sex was a tertiary factor in these studies and thus not fully explored.

To maximize the sample size, I used data from the first level of the video game across four previously published experiments in which this data was not analyzed (Young and Nguyen, 2009; Young et al., 2011a,b). I only used data from experiments and conditions in which the training level was identical – choosing among three targets in each of seven groups, no delays, 100% likelihood of the weapon working, and no stress.

## MATERIALS AND METHODS

### PARTICIPANTS

A total of 213 introductory psychology students (105 women and 108 men) at Southern Illinois University at Carbondale received course credit for their voluntary participation.

### GAME ENVIRONMENT AND DESIGN

The Torque Game Engine (obtained from www.garagegames.com) was adapted as the platform for game development. Torque’s first-person-shooter starter kit involves a rich world containing hills, mountains, buildings, lakes, a crossbow that shoots exploding projectiles, and orcs – monster-like characters. The game contained seven separate regions with each region populated by three visually identical orcs. For simplicity, the orcs were stationary and oriented toward a target region (e.g., a building) that the player was directed to protect. Each group of three orcs was oriented toward a different building to maintain a distinctive target trio. Every 4 s (on average), each orc fired its weapon (an orc’s firing was noticeable from the recoil of the weapon and an audible click, although it may or may not have produced an explosion). Specifically, every 4 s each orc was programmed to fire once within the next 3 s with the precise delay chosen from a (0, 3 s) uniform distribution. For the enemy orc, its firing was immediately followed by the explosion.

The player’s task was to identify the enemy orc that was producing the explosions and destroy it. Destruction of a single orc required eight shots (a fixed-ratio 8 or FR8 schedule), because our pilot studies revealed that participants showed greater discernment under these conditions because poor choices would lengthen the game. For a video clip showing a participant observing a trio, destroying a target (i.e., making a choice), and observing the consequences of their choice, see “Video game clip of basic causal task” at http://www.k-state.edu/psych/research/young/suppmaterial.html This clip also shows a bird’s eye view of the entire game region.

### PROCEDURE

The participants were seated at one of four identically configured 1.25 GHz Mac Mini computers. The participant received specific verbal instructions, including a description of the task, using a screenshot of what they would see once the experiment was started. The instructions included the following:

Today you will be participating in a computer experiment. You will be placed in a virtual village being attacked by sets of orcs. The orcs will be firing timed grenades. Only one of the orcs has a working weapon; the others are duds. Your task is to identify and destroy the orc causing the damage, the enemy, without shooting the duds. If you choose the right orc, the explosions will stop and the blue bar will drop on the screen. If you choose the wrong orc, the explosions will continue and the blue bar will persist. Continue firing on one of the remaining orcs that is causing the damage until you discern the enemy.

In addition to the task instructions, participants were advised how to navigate within the environment. When the participants indicated an understanding of the procedure, the experimenter started each of the programs.

Once a participant had destroyed all of the enemies, the video game ended. After the period of behavior described in the present study, participants performed other gaming decisions described elsewhere (Young and Nguyen, 2009; Young et al., 2011a,b). After finishing the game, the participants completed a demographic questionnaire asking their sex, self-rated video game experience during elementary school, middle school, high school, and college (Likert scale with 0 indicating none, 6 indicating daily), and types of video games that they play. Because a principal components analysis revealed no distinct roles for periods of experience, I summed across the four periods of experience.

## RESULTS

Both choice accuracy and the time elapsed between the previous correct decision and the first choice at the next trio were examined. Therefore, latency includes travel time and observation time. Unfortunately, the contamination of latencies with travel time is unavoidable with the current design because people can observe the targets firing while traveling between the target groups. To prevent this contamination, prior research has occasionally placed the targets within a hut to prevent observation while traveling and to begin recording latency upon entry into the hut (Nguyen et al., 2010; Young et al., 2011a). Although the latencies were obviously much shorter when travel time was removed, the relationship between the predictors and latency was maintained regardless of the inclusion of travel time (including sex differences). These results suggest that travel time is relatively constant across conditions and participants. However, these previous studies always assessed performance after the first level. It is quite possible that participants would show rapid decreases in travel time as they gain familiarity with the game environment throughout the first level thus making it difficult to distinguish decreases in latency due to more efficient travel or shorter observation or decision times. An alternative analysis will be conducted on a subset of the participants to attempt to address this issue.

I used a model comparison approach in the data analysis – the simplest model of theoretical interest was chosen. Thus, terms that did not reach statistical significance were eliminated and higher-order interactions involving the individual differences variables were not examined. Because prior work with the gaming platform often documented an association between the dependent variables (accuracy and latency) and sex and self-reported amount of video game experience, only those variables were examined.

All analyses were conducted using generalized linear mixed effects modeling (also known as generalized multilevel modeling). This approach is the preferred method of analyzing repeated measures data involving non-normal error distributions (Gelman and Hill, 2006; Bolker et al., 2008; Dixon, 2008). Because this model selection approach introduces additional degrees of freedom (Dixon, 2013), I used the more conservative Bayesian information criterion (BIC) as a model selection metric.

### ACCURACY

Analyses of accuracy were performed on trial-level data and thus required the specification of a binomial distribution (i.e., I performed repeated measures logistic regression). I examined two classes of models, those using order and experience as linear predictors and those exploring log transformations of order and experience. A log transformation of order was considered because behavioral change was expected to be greater for the first few decisions, and a log transformation of experience was considered because the experience variable was highly skewed (very few participants reported high amounts of video game play). Adding self-reported experience to the models (linear or log-transformed) produced poorer models and thus it was not included. Using a log transformation of choice order produced a model that was 7.3 times more likely to have produced the data than the model assuming a linear relationship and thus a log relationship was assumed.

**FIGURE 1:**
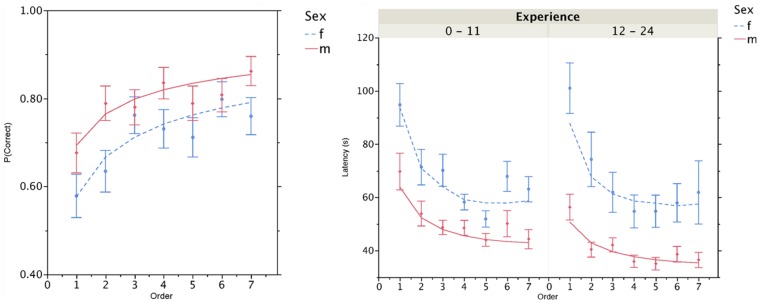
**(A)** Increase in accuracy as a function of the order in which a decision was made, shown separately for men and women. **(B)** Decrease in latency as a function of decision order, shown separately for men and women and self-related experience playing video games. Experience was analyzed as a continuous variable (0–24) but has been dichotomized here into low (0–11) and high (12–24) for presentation purposes. All lines are those derived from the best fitting statistical model.

The best fitting model only included main effects of order and sex; the model fit is shown in the left side of **Figure 1**. The analysis revealed an increase in accuracy as a function of order (*b* = 0.61, *z* = 6.04, *p* < 0.01) and higher accuracy for men than for women (84% vs. 75%, *z* = 2.92, *p* < 0.01).

### LATENCY

Analyses of latency were performed on trial-level data and with the specification of a Gamma error distribution due to the skewed distribution of latencies (i.e., I performed repeated measures gamma regression). I again examined two classes of models, those using order and experience as linear predictors and those exploring log transformations of order and experience. Using a log transformation of choice order and assuming a linear relationship with experience produced the best model.

The best fitting model only included main effects of order, sex, and self-reported amount of video game experience; the model fit is shown in the right side of **Figure 1**. The analysis revealed a decrease in latency as a function of order (*b* = 0.0051, *z* = 10.91, *p* < 0.01), shorter latencies for men than for women (43.8 s vs. 66.5 s, *z* = 3.82, *p* < 0.01), and a decrease in latency as a function of prior video game experience (*b* = 0.0003, *z* = 3.36, *p* < 0.01). Note that gamma regression uses an inverse link function thus slope estimates (betas) were positive because larger order values or more experience result in faster times to the first choice.

There was a 22.7 s difference in mean latencies for men and women at each decision point. This difference could be due to women spending more time in travel between target trios or taking more time to observe the causal relationship once they have arrived at the targets. In trying to distinguish between these two sources, I was able to obtain data on the navigation of the environment for 132 of the participants (this data was not retained for the other participants). The program stored player position every 1 s during game play. This data was used to create two measures, the total amount of time in movement and the total amount of time sitting still. Although participants could observe while moving, these two measures may serve as a proxy for distinguishing differences in navigation efficiency from differences in observation time. The time spent idle includes the eight shots it took to destroy a target (≈8–10 s), but this duration is consistent across conditions and participants.

In examining the average time spent moving between decision points during the first level of the game, women took 7 s longer than men [31 s vs. 24 s, *t*(131) = 3.01, *p* < 0.01]. Interestingly, this difference was much smaller for subsequent levels that were not part of this study (3, 2, and 1 s, for levels 2 through 4). Regardless, this analysis suggests that part of the 23 s latency difference is due to women taking longer to travel between target trios. Although this result might be of interest for other lines of research, it has little bearing on our understanding of sex differences in causal inference.

More interestingly, the analysis of time spent idle in the game revealed that women were idle 17 s longer than men during the initial level [44 s vs. 27 s, *t*(131) = 6.47, *p* < 0.01]. This difference remained quite large for subsequent levels that were not part of this study (15, 10, and 9 s, for levels 2 through 4). Although the analyses of movement time were performed on a subset of the participants, these results suggest that the majority of the observed latency differences between men and women (~23 s, see **Figure 1**) was due to observation time rather than navigation efficiency (~17 s vs. ~7 s).

### EVIDENCE OF PERCEIVED VERSUS INFERRED CAUSATION

When people judge an interaction to be causal without the need to observe the relationship multiple times and can do so with high accuracy, the process is often described as a demonstration of causal perception (Leslie, 1986; Scholl and Tremoulet, 2000). In contrast, the need for repeated observation or an increase in causal judgment accuracy over time suggests the action of an inferential process based on evidence accumulation (Shanks and Dickinson, 1987; Wasserman, 1990; Young, 1995). Although the ability to make any firm claims regarding perception versus inference is limited by the absence of data on exactly how many times a player observed each relationship (a shortcoming due to the use of a free operant task), I examined data that might provide some indication in favor of perception versus inference. If most participants demonstrated an immediate recognition of the causal relationship between the cause and its effect, then accuracy should be perfect for those participants; the learning effects may be an averaging artifact produced by a small number of observers who must adapt to the environment. In contrast, if most participants are making mistakes despite their opportunity to observe multiple shots by each candidate, this is inconsistent with an automatic process thus suggesting that an effortful inference is required.

**FIGURE 2:**
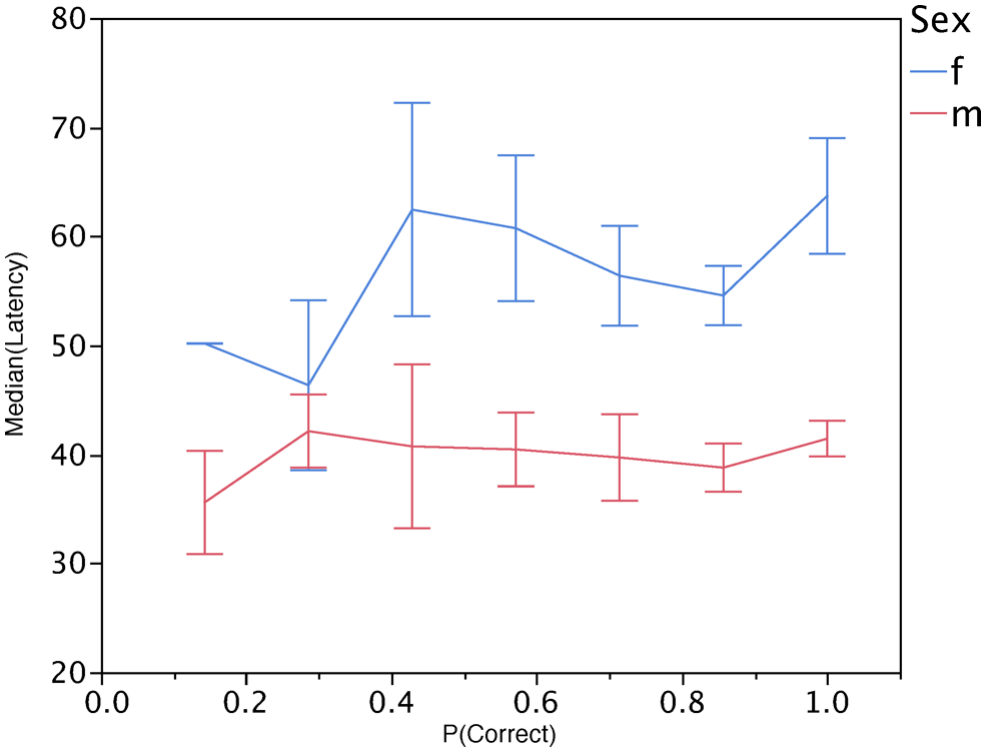
**Median latency to make a decision for individual participants with each level of accuracy across the seven decisions**.

When examining the accuracy of individual performance at all seven choice points, 17 of the 104 women were perfect and 36 of the 108 men were. Because the small number of perfect judgers could be affected by an initial unfamiliarity with the game, I re-examined the issue after the first three decisions points – 42 women and 62 men were perfect on the last four decisions indicating that a sizeable number were still making mistakes despite their ability to extend their observation time to ensure greater accuracy. Furthermore, there was no evidence that participants who took longer to make their decisions were more likely to be correct. **Figure 2** shows the median latencies for each individual as a function of their accuracy. The only obvious relationship evident in the figure is the longer latencies for women at every level of accuracy, even among those who achieved perfect performance. In sum, the judgment is not easy, automatic, and consistent in the same manner as perceptions of direct causal relationships like the launching effect.

## GENERAL DISCUSSION

Although the causal relationship being observed in the game was quite simple, involving no delays or uncertainties, most participants showed evidence of imperfect accuracy and often observed multiple instances of the relationship before making a decision (averaging about five shots for men and nine shots for women after removing time related to movement and shooting). This need to observe could be driven by the possibility that the candidate causes can occasionally fire in very close temporal proximity thus confounding the determination of causation, but it also could be driven by the unfamiliarity of the causal relation. Unlike the physical interaction of objects being mimicked in studies of Michotte’s launching effect, the relationship between the firing of a video-game weapon and a distal explosion certainly does not carry the same experiential basis as billiard ball causation. Indeed, our adoption of a video game platform was partially motivated by the desire to study causal relations that must be learned through experience, thus comparable to other environments in which causality is not directly perceived like medicine, chemistry, education, or technology. When people interact with modern technological environments with invisible forces and mechanisms and causality at a distance, the causal dynamics are quite unlike those found in the natural environment in which our species evolved. Even a person born a millennium ago would find the relationships produced by modern technology more akin to magic than arising due to physical laws.

### SEX DIFFERENCES

Discovering causal relationships appears to be a basic process which, when mastered, provides a significant advantage to members of every species. Individual members of a species, however, may differ in the method of identifying a causal relationship, the time it takes to do so, and the contexts in which it is done efficiently. We observed substantial differences between men and women in accuracy and observation time within the context of a video game task. Differential experience with video games explains part of the latency differences among participants (see right side of **Figure 1**), but a large sex difference remained after controlling for experience and persisted even after gaining experience within the game. Prior causal decision making studies involving the same gaming platform (Young and Nguyen, 2009; Young et al., 2011a,b) only examined performance after the initial seven choices to decrease the effects of unfamiliarity with game controls. These studies occasionally revealed that differences in accuracy between women and men disappeared when controlling for self-reported prior gaming experience, but latency differences persisted and remained substantial. The present analysis of early behavior in the game is consistent with these previously published results.

Given the consistent sex differences observed in our causal learning task, why do they occur? When we first began using a game-based methodology, we suspected that men and women might differ due to their divergent experiences playing video games. However, even when this factor has been statistically controlled, the large differences in latency remain. Furthermore, impulsivity experiments involving the same gaming environment fail to show sex differences (e.g., Young et al., 2011c, 2013). However, the trappings of a first-person-shooter video game may be off-putting for a subset of our participants, especially women who might have been socialized to dislike male-stereotyped video games (Lucas and Sherry, 2004).

Alternatively, a speed-accuracy tradeoff could be driving the difference if women are waiting longer to increase accuracy due to aversion to risk (Kimura, 2000; Frederick, 2005), but there was no evidence of such a tradeoff – women observed much longer before deciding, but waiting did not result in higher decision accuracy. Another possibility is that the visual and spatial nature of the task might be producing a sex difference similar to that observed in certain other spatial tasks (Kimura, 2000). Although a handful of published studies involving causal decision making tasks report a small male advantage (Shaklee and Hall, 1983; Wasserman et al., 1983; Wasserman and Neunaber, 1986; Nguyen et al., 2010; Young et al., 2011a), most studies do not include sex as a predictor thus making it difficult to appeal to a task-based explanation. I uncovered only one report of a sex difference in a causal perception task involving adults, with men more likely to judge a relationship as causal (Tschacher and Kupper, 2006).

Regardless of their source, the existence of these differences early in the gaming task reveals that men and women differ in their success at discovering the causal relationships in this complex environment. These individual differences are likely to be significantly reduced with extensive training and experience, but the degree to which they can be eliminated remains an open question and depends on the source of the performance disparity across sexes.

### CAUSAL DISCOVERY IN COMPLEX ENVIRONMENTS

Other than, perhaps, the simplest forms of physical causality involving contact (Michotte, 1946/1963; White, 1988), causal relations must be inferred as a function of experience. The presence of spatial and temporal contiguity and a high contingency enables strong prediction of the consequences of certain actions or events. However, even when the cues to causality are all present, complex environments can confound these cues in the presence of multiple candidate causes. When two candidates co-occur or nearly so, it is not possible to easily distinguish between them as causes of an outcome after a single observation. Therefore, even in otherwise ideal conditions people sometimes need to observe a series of events multiple times to gain sufficient confidence in their judgment to identify a cause. Interestingly, people in the game were learning to engage in this causal discovery process more efficiently as they performed the task. Not only did their accuracy increase with each decision, but their latencies decreased due to more efficient travel and, likely, shorter observation times (**Figure 1**). Given the nature of many causal decision making environments, from the diagnosis of disease to the tracking of information on device control panels, a greater understanding of both the genesis and application of causal knowledge in the face of complexity will determine whether the standard cues to causality suffice or if new processes emerge (Young, 2012).

## Conflict of Interest Statement

The author declares that the research was conducted in the absence of any commercial or financial relationships that could be construed as a potential conflict of interest.
